# HIV-Associated Cryptococcal Meningitis: A Call for Action for New Treatment Options

**DOI:** 10.3390/therapeutics3020009

**Published:** 2026-03-31

**Authors:** Samuel Okurut, David B. Meya

**Affiliations:** 1 Research Department, Infectious Diseases Institute, Makerere University, Kampala P. O. Box 22418, Uganda; 2 Division of Infectious Diseases and International Medicine, Department of Medicine, University of Minnesota, Minneapolis, MN 55455, USA; 3 Department of Medicine, School of Medicine, College of Health Sciences, Makerere University, Kampala P. O. Box 7072, Uganda

**Keywords:** cryptococcal meningitis, clinical trials, antifungal therapy, human immunodeficiency virus, mortality improved regimen, survival

## Abstract

Cryptococcal meningitis occurs in 62% of persons with HIV-associated meningitis, making Cryptococcus an important cause of meningitis among adults with advanced HIV disease in regions with elevated prevalence of HIV. Despite efforts to advance treatment, the in-hospital death rate of 19% remains unprecedentedly high. Aggregate published clinical trial data evaluating cryptococcosis treatment with survival as the primary endpoint show a significant reduction in the proportion of survivors from diagnosis to 88.5% at 2 weeks of treatment and further to 74% survival at 10 weeks of follow-up (*p* = 0.001). Disease complications concomitant with unveiling symptoms and reoccurrence of fungal infections, deferment in treatment, and high prevalence of other comorbidities increase the risk of individuals succumbing to cryptococcal meningitis. Among clinical trials of cryptococcal meningitis, the World Health Organization-recommended standard of care was used to randomize participants to the control trial arm. The proportion of participants surviving in the trial was not statistically different between trial randomization arms. In summary, high in-hospital death rates and continued participants’ deterioration post-hospital discharge are challenges for evidence-based new therapies seeking to improve outcomes.

## 1. Introduction

Cryptococcal meningitis (CM) is the top cause of meningitis in people with advanced HIV disease, contributing to a reduction in hospital survival from the time of admission (100% persons at risk at admission) to 85% rate of survivors (inter-quartile range, (IQR); 87–76% survival). Among infections causing meningitis, tuberculosis meningitis, at 21% of infections, is the second most common cause of meningitis with HIV co-infection [1]. Severe HIV-associated immunodeficiency with advanced HIV disease with CD4 T cells <100 cells/microliter remains the significant clinical marker of exposure, disease severity, and increased risk of death [2]. In low-income settings, advanced HIV infection/disease is primarily associated with the risk of cryptococcosis [3]. In the Western world, the high prevalence of multiple comorbidities contributes to the great risk associated with cryptococcosis [4,5]. Among prevalent risks associated with cryptococcal meningitis in the Western world include: HIV infection (44.4%), overuse of corticosteroid treatment (28.3%), malignancies and cancers (25.5%), smoking (20.0%), organ transplants (18.9%), and underlying respiratory disease conditions (18.2%) [4,5]. Despite attempts to advance treatment, high death rates are a persistent concern, with mortality rising to 45% by 10 weeks of diagnosis and follow-up of treatment, especially in resource-limited settings [1,6–8]. Speedy diagnosis and treatment initiation are difficult. The disease presentations among immunocompromised individuals at risk of other high-prevalent existing co-infections with HIV complicate early diagnosis and treatment [6,9–11].

Objectively, global mortality rates (19%) from cryptococcal meningitis are lower than the 30% death rate observed in the developed world [4,5]. The global cryptococcal meningitis death rate of 19% is lower than the 28–33% death rate in largely Caucasian (67–73% of patients), adult (age: 40–70 years old), and male (79–82%) participants in the Western world [4,5]. Despite comorbidities and HIV advanced disease being linked to the incidence of cryptococcosis infection and disease severity, the pathogenesis mechanisms, factors associated with the adverse outcome, and the extent of disability among patients are similar, irrespective of the region. The commonly observed aberrant outcomes include: hearing deficits (16%), frailty (40%), neurocognitive deficits (33%), hydrocephalus needing mechanical CSF drainage (7.4%), stroke (33%), and recurrent infections (23%) [4]. The finding that 23% of patients with cryptococcal meningitis in the United States cohorts experienced recurrence of cryptococcal meningitis, yet with the high risk of death at readmission [3,4,9,11], suggests the high disease severity index in this population, possibly due to late presentation, delay in diagnosis and treatment, severe immune deficiency, and high prevalence of multiple comorbidities. Apart from high prevalence of co-morbidities and other factors associated with bad outcomes, persons at greater risk of succumbing to death often have low CSF immune response and severely deficient T cell response [12].

Antifungal therapy may be effective in suppressing fungal infection, but death rates remain alarmingly high. Persistent high mortality rates could be independent of the efficacy of antifungal agents and dependent on host-associated immunological and metabolomic deficits [13]. Therefore, high mortality rates associated with cryptococcal meningitis are a conundrum impacted by the severity of the disease, possible late diagnosis with the high fungal burden, severe immunodeficiency, and other comorbidities rather than solely on the inefficiency of the antifungal treatment. Therefore, some patients at risk of death could benefit from a possible host-directed combined treatment-modifying intervention to improve survival. Additionally, whether fungi persist in disseminated deep tissue niches and cause relapse of cryptococcal meningitis is not known, especially in human cohort studies. Therefore, whether CSF fungal clearance is a proxy for fungal clearance in disseminated tissues is an area requiring further research [13].

## 2. Materials and Methods

### 2.1. Design, Purpose, and Approach

We consolidated the literature and important observations from peer-reviewed HIV-associated cryptococcal meningitis clinical trials where treatment to improve outcomes, especially survival, was assessed as the major research outcome at 2 weeks and 10 weeks of follow-up after diagnosis and clinical trial randomization.

**Figure 1.**
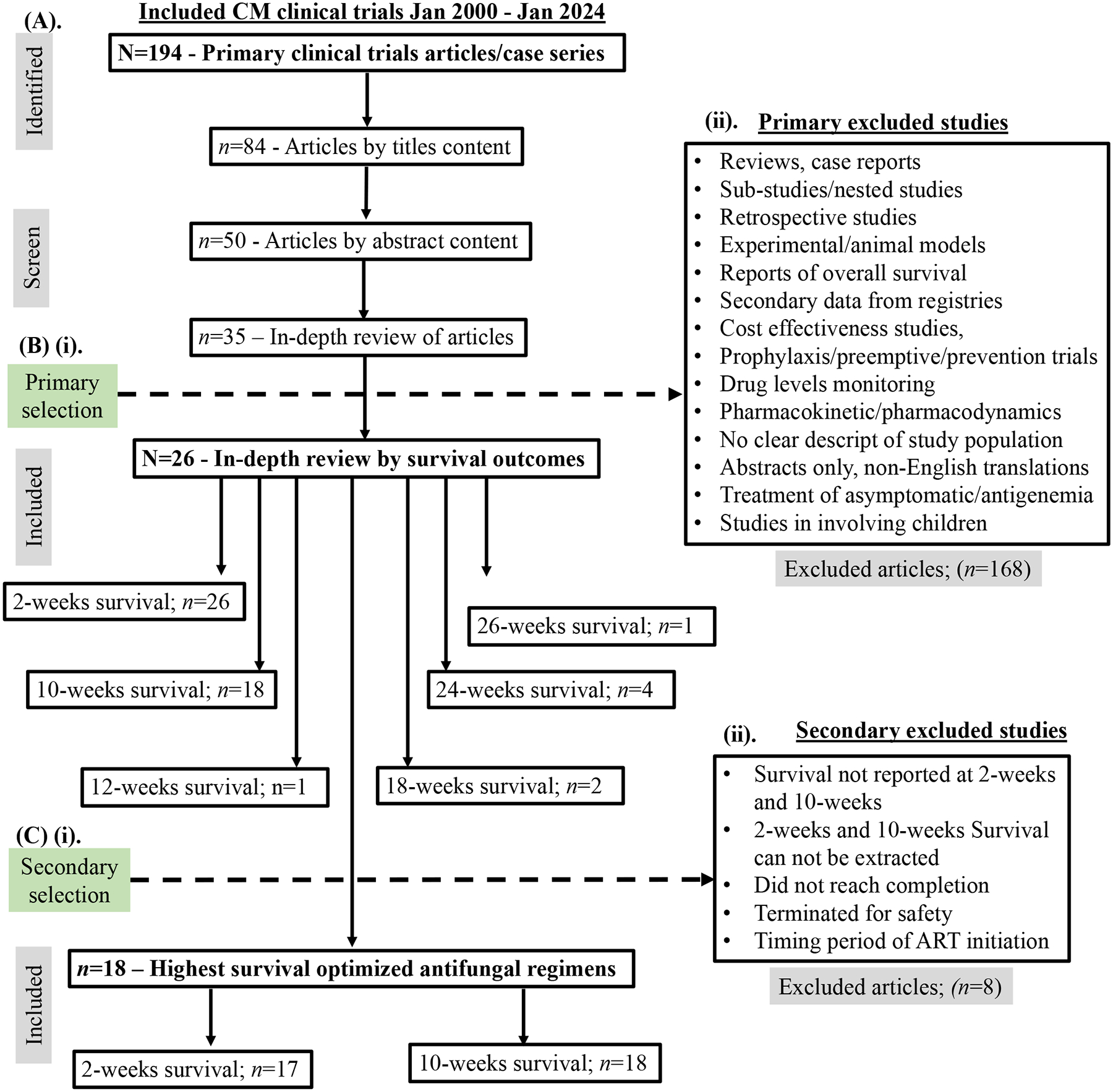
The approach taken to include or exclude articles that contributed data to the study. (**A**) Articles found in the PubMed database using “cryptococcal meningitis Clinical Trials” free word search customized to a period between January 2000 and January 2024; (**B**)(i) articles reporting survival as a definite time-bound outcome; (**B**)(ii) sample of issues leading to exclusion of article that did not meet inclusion criteria; (**C**)(i) articles reporting survival by 2- and/or 10-week survival; (**C**)(ii) articles excluded because survival was reported outside the 2- or 10-week follow-up window. n – represents the sample size.

We searched the PubMed-indexed peer-reviewed literature for major cryptococcal meningitis clinical trials published between January 2000 and January 2024 by the “Cryptococcal meningitis Clinical Trials” MESH phrase. The detailed approach undertaken to consolidate the data is highlighted in Figure 1. The objective was to establish and strengthen evidence-based interventions, regimens, and knowledge from cryptococcal meningitis clinical trials in the past 2.5 decades of intensive research. The target was primary research outputs, especially from tertiary hospitals and state-of-the-art facilities with controlled and protocolized intervention.

### 2.2. Inclusion and Exclusion Criteria

The objective was survival data from cryptococcal meningitis human trials; (N = 194) online articles were available (Figure 1A). The primary selection and inclusion criteria were all parent trials that reported survival as the primary endpoint at definite time points (*n* = 26) (Figure 1B(i)). Studies not meeting the primary inclusion criteria (*n* = 168) were excluded from the meta-analysis (Figure 1B(ii)).

Further secondary sections or inclusion criteria were all trials reporting survival at 2 or 10 weeks. (*n* = 18) (Figure 1C(i)). Studies not meeting the secondary inclusion criteria (*n* = 8) were excluded from the 2-week and 10-week survival meta-analysis (Figure 1C(ii)).

### 2.3. Meta-Analysis

**Figure 2.**
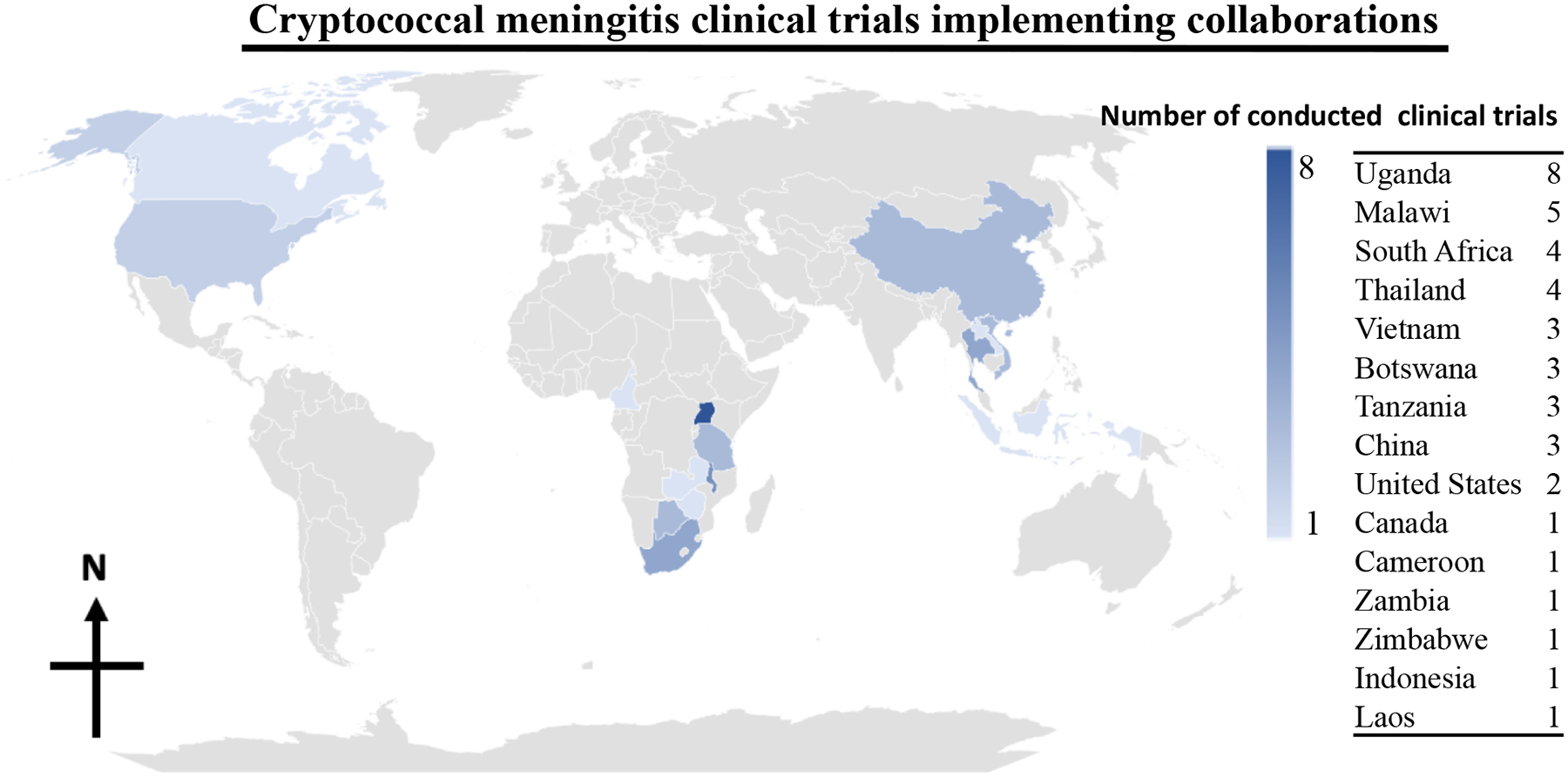
Global distribution of cryptococcal meningitis clinical trials sites reported among 18 peer-reviewed articles contributing to the dataset [14–31].

Summary data was compiled and tabulated in Excel. Data visualization was displayed in graphs, line graphs, bar charts, and a global map representing countries where trials were implemented (Figure 2). Simple comparative statistics were performed using the Mann–Whitney *t*-test.

## 3. Results

### 3.1. Therapeutic Monitoring of Efficacy of Cryptococcal Meningitis Antifungal Therapy and Trials Endpoints

The primary included studies (*n* = 26) reported survival as a primary outcome at different time points. All studies reported survival at 2 weeks, while 69% of the studies (18 of 26 studies) reported survival at 10 weeks. Eight studies: one reported survival at 12 weeks, two reported survival at 18 weeks, four reported survival at 24 weeks, and one study reported survival at 26 weeks.

### 3.2. Progress in the Development and Testing of Antifungal Treatments to Improve Outcomes in Cryptococcal Meningitis

In an attempt to improve the outcome of patients diagnosed with HIV-associated cryptococcal meningitis, repurposed antifungal compounds and formulations have increasingly been evaluated. The clinical trials are concentrated in epicentres and hotspot regions with high prevalence of HIV (Figure 2). Since the major outbreak of HIV 4 decades ago, cryptococcal meningitis has remained a major opportunistic infection associated with advanced HIV disease in areas of high HIV prevalence including Uganda, Malawi, South Africa, and Thailand. Other countries participated in 1–3 clinical trials. Hence, cryptococcal meningitis and associated risk factors for disease manifestation and outcomes are a persistent setback for treatment advances to improve the quality of life among people living with HIV in regions with high prevalence of HIV.

### 3.3. Decades of Efforts to Improve Treatment of Cryptococcal Meningitis

**Figure 3,:**
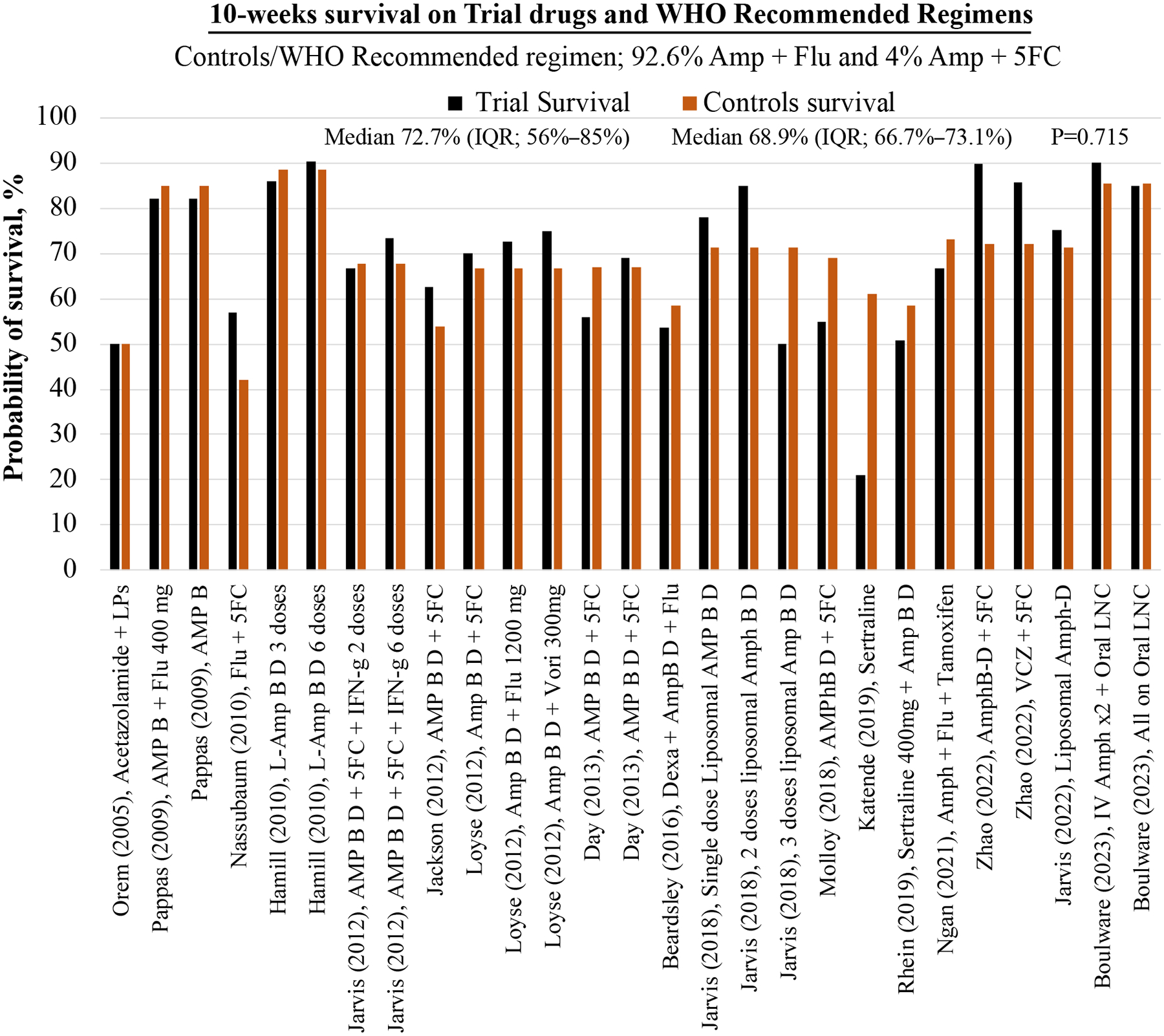
10-week survival by randomization arms in the inductive trial drugs arm or on the control trial arm (standard treatment) [14–17,19–28,31–37].. (N = 3434 participants), (*n* = 1710 trial arms randomized cases, and *n* = 1724 control randomized arm were included, randomized in a 1:1 ratio) among 18 studies with completed trial data. Flu—fluconazole, 5FC—flucytosine, AmB—amphotericin B, L-AmB—liposomal amphotericin B deoxycholate, LPs—lumbar punctures, IFN-γ—interferon gamma, mg—milligram, Vori—voriconazole, VCZ—voriconazole, IV—intravenous, LNC—liposomal amphotericin B deoxycholate lipid encapsulated nanocrystals, dexa—dexamethasone, x2—two times. Comparative paired analysis *p*-value (*p* = 0.715) was not statistically significant.

Among clinical trial treatment randomizations, 95% of the trials had standard cryptococcal meningitis treatment in the control arm as a comparative trial regimen (Figure 3). Among cryptococcal meningitis treatment options, liposomal amphotericin B (Ambisome) or liposomal amphotericin B deoxycholate formulations in combination with Fluconazole or Flucytosine were used for the induction treatment of cryptococcal meningitis. Fluconazole or flucytosine is used for consolidation treatment of cryptococcal meningitis. Fluconazole monotherapy is used for maintenance therapy to prevent possible relapse of cryptococcal meningitis.

**Figure 4,:**
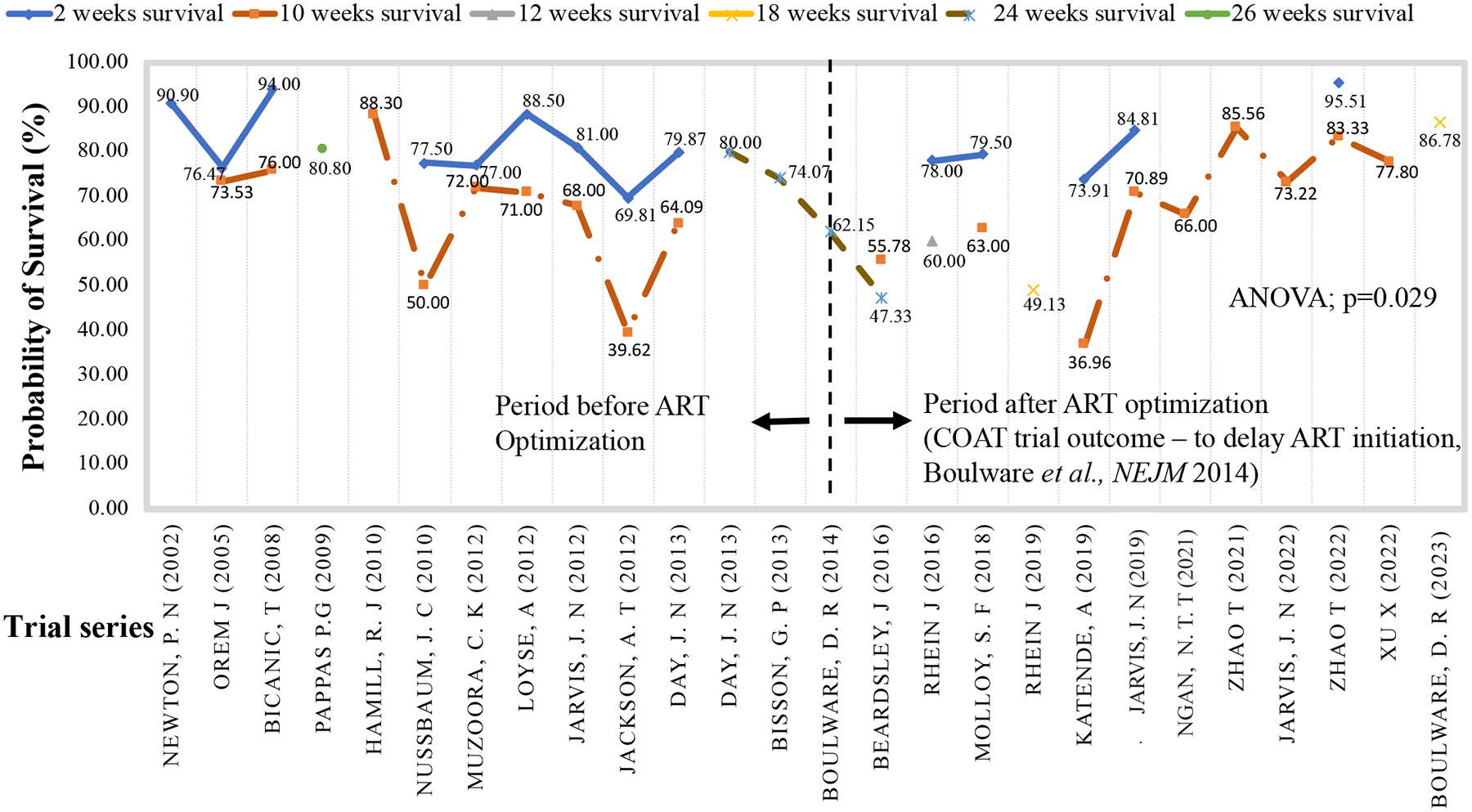
Survival among participants enrolled in cryptococcal meningitis treatment and survival improvement clinical trials conducted between January 2000 and January 2024 [14–28,31–38] ART—antiretroviral therapy, COAT—cryptococcal optimal antiretroviral therapy timing, ANOVA—analysis of variance across reported survival categories labelled above the figure. The comparative ANOVA *p*-value (*p* = 0.029) was statistically significant.

Among clinical trials, amphotericin B and fluconazole were used in 92% of trials (25/27 trials) as the induction regimen. Amphotericin B and fluconazole were used in only 4% of trials (1/27 trials) as an induction regimen for treatment of cryptococcal meningitis (Figure 4 and Table 1). In either trial randomization arm, no significant differences were observed between the trial arms (*p* = 0.715) (Figure 3 and Table 1).

### 3.4. Cryptococcal Meningitis Outcomes with Deferred Antiretroviral Therapy

Outcomes among trials optimizing treatment for cryptococcal meningitis differed significantly between the primary and secondary endpoints (*p* = 0.029) (Figure 4). However, there was no major observable difference in outcomes with antiretroviral therapy optimization among trials related endpoints. Though cryptococcal antimicrobial compounds predominated in the era of cryptococcal meningitis with advanced HIV disease, other trials using corticosteroids and interferon molecules were designed to advance formulations and clinical interventions for host-directed adjunctive therapies in combination with antifungals.

Generally, few clinical trials reported follow-up observations beyond 10 weeks, perhaps because of the prohibitively high-cost implications in protocol design and implementation. The limited period of observations limits the understanding of the long-term effects of cryptococcal meningitis. Therefore, extrapolation of findings beyond 10 weeks of follow-up is a challenge.

### 3.5. High Survival Rates with New Cryptococcal Meningitis Antifungal Formulations

**Figure 5.**
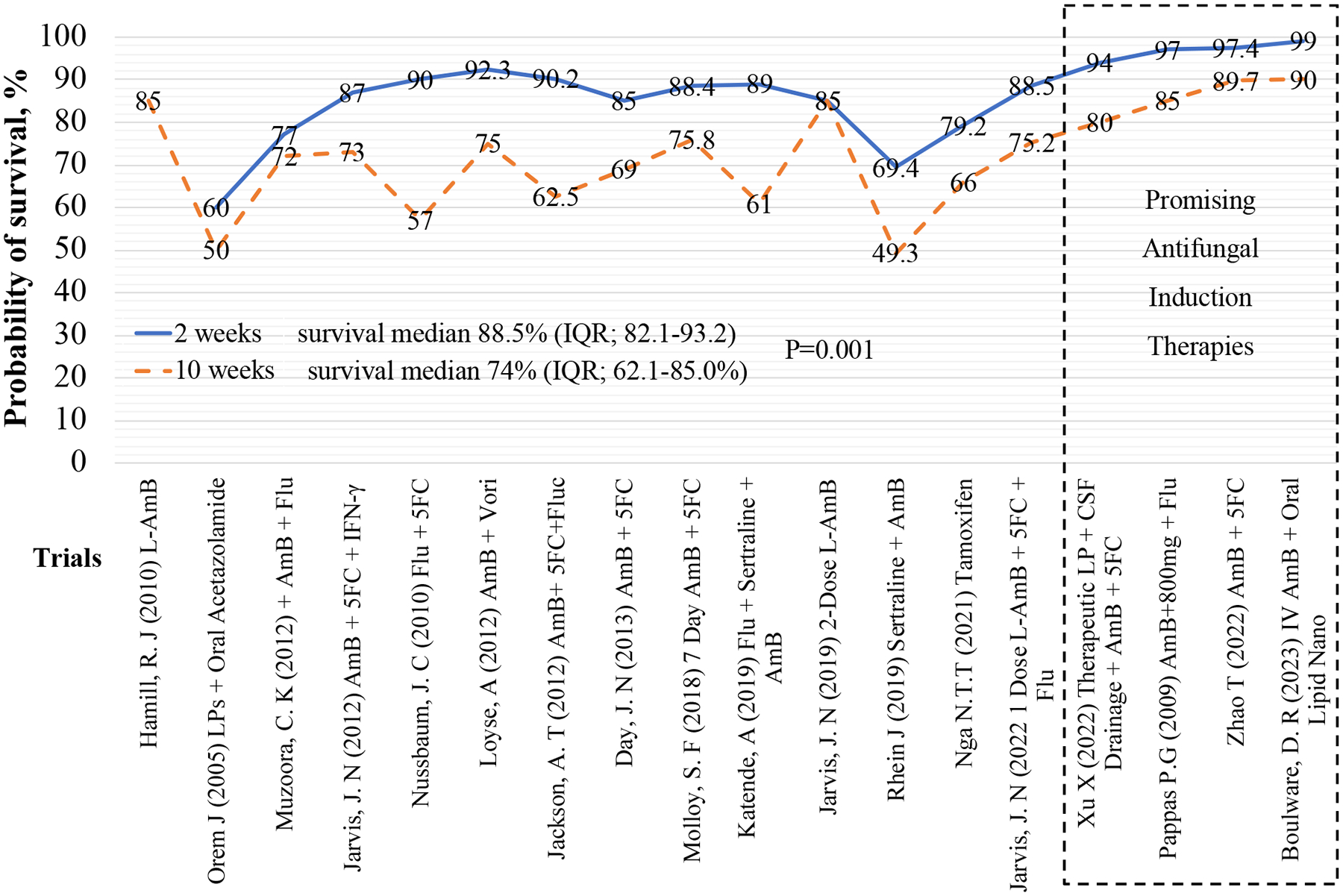
Outcomes among 18 studies reporting 2-week and/or 10-week survival with treatment of cryptococcal meningitis. The participants contributing data were [14–28,31–37]. Flu—fluconazole, 5FC—flucytosine, L-Amp—liposomal amphotericin B deoxy-cholate, VCZ—voriconazole, Vori—voriconazole. Crypto-Dexa—cryptococcal dexamethasone. Adjunctive therapies include dexamethasone, sertraline, tamoxifen, and acetazolamide. The comparative *p*-value *p* = 0.001 was statistically significant.

Consistently, the proportion of survivors significantly declined from enrolled individuals with cryptococcal meningitis to 2-week survival at 89% (IQR: 93–82% survival) (Figure 5). Subsequently, host survival continued to decline from 2-week reported outcomes to 74% survival (IQR: 62–85% survival) at 10 weeks of follow-up (*p* = 0.001).

However, four seminal clinical trials and antifungal formulations for the treatment of cryptococcal meningitis made it to the top in phase IIb trials. The fourth cryptococcal meningitis treatment with the highest yield of survivors had 99% two-week survival and 90% 10-week survival using oral lipid-bound amphotericin B deoxycholate compounds. The second antifungal formulation with better yield of survivors was liposomal amphotericin B deoxycholate. Flucytosine used as an inductive regimen for the treatment of cryptococcal meningitis yielded 97% in-hospital two-week survival and 90% 10-week follow-up survival. The third best antifungal regimen was liposomal amphotericin B and fluconazole, which yielded 97% in-hospital two-week survival and 85% 10-week survival at follow-up.

The fourth, yet non-drug related add-on intervention with antifungal therapy, was the implementation of two or more therapeutic serial lumbar punctures and CSF drainage for additional diagnostics, monitoring, and managing intracranial pressure. The addition of serial therapeutic lumbar punctures and CSF drainage significantly improved both the 2-week and 10-week patients’ survival outcomes, yielding 94% 2-week survival (IQR: 90–99% survival) and 80% survival (IQR: 76–87% survival) at 10-week follow-up.

## 4. Discussion

The major driver of efforts to improve treatment and antifungal formulations revolves around the low survival rate of patients on cryptococcal meningitis antifungal therapy [13]. Key observations show the significant risk of death among participants diagnosed with cryptococcal meningitis. Consistently, the number of patients dying from cryptococcal meningitis significantly increases from 2 weeks to 10 weeks of observation despite deferment of antiretroviral therapy. The number of patients dying from cryptococcal meningitis is more likely associated with the possible high prevalence of treatment-modifying co-infections and comorbidities with advanced HIV disease than with the efficacy of antifungal treatment. Irrespective of the clinical trial, the standard of care for cryptococcal meningitis formed the backbone of the trial regimens. Though survival continued to decline in the 10 weeks of follow-up, there were limited reported findings beyond 10 weeks that limit generalization of results to long-term outcomes with cryptococcal meningitis. Among clinical trial regimens, three seminal antifungal formulations and one non-drug clinical therapeutic intervention contributed the highest life years saved with cryptococcal meningitis treatment options. Among cryptococcal meningitis clinical trials, Uganda led most trials (eight studies), followed by Malawi (five trials), with South Africa and Thailand completing four trials.

Reliance on rapid antimicrobial fungal killing is a mechanism used to determine the efficacy of antifungals. Additionally, clinical improvement is used to assess antimicrobial effectiveness, as presenting symptoms clear and fungal culture turns negative [39]. The key kinetics of the antifungal efficacy monitoring approach depend on the effects of antifungal treatment on fungal clearance and absence of fungal growth on CSF culture [13]. This treatment advance considers the infectious disease management approach of rapid killing of microbes, faster clearance of pathogens, and negative culture conversion as an emergent intervention to alleviate the suffering of patients and improve treatment outcomes [40]. Reliance solely on antimicrobial use in advanced HIV disease, without considerations for host-directed therapies for existing or unmasking co-infection, comorbidities, and immune suppression, can compound the efficacy of antifungals in improving trial outcomes. Consistently, individuals with poorer outcomes have low immune response and other significant clinical and neurological deficits requiring possible host-directed corrective and/or treatment-modifying therapies [41–43]. Moreover, the variety of viral co-infections and other comorbidities are frequently diagnosed and managed in the cryptococcal meningitis cohort [8]. These factors together delay and complicate the diagnosis and treatment of HIV-associated cryptococcal meningitis and have a confounder effect on the assessment of efficacy of antifungals in use in influencing poor outcomes.

Among antifungal agents in use, a combination of antifungal compounds is superior in antimicrobial activity compared to monotherapies [44]. Among antifungals in use, old antifungal formulations with amphotericin B, discovered in [45], and flucytosine, both discovered over 7 decades ago [46], and fluconazole, discovered over 5 decades ago [47], formed the core compounds in cryptococcal meningitis treatment optimization trials. The cryptococcal meningitis clinical trial formulations included intravenous liposomal amphotericin B, amphotericin B deoxycholate, or the oral lipid nanocrystal formulation of amphotericin B deoxycholate (0.7–1 milligram per kilogram of body weight). Fluconazole 800 milligrams–1200 milligrams daily dose or flucytosine 100 milligrams per kilogram of body weight ingested four times daily was used as an inductive therapy for the first 7–14 days. The inductive antifungal treatment dosing period or dose escalation is dependent on the outcomes of fungal clearance in CSF cultures. The 800 milligrams daily dose of Fluconazole for 8 weeks is used as consolidation treatment. The antifungal maintenance period is achieved with a 200 milligram daily dose of fluconazole for further prevention of cryptococcal meningitis relapse for at least a year [37,48].

Participants’ survival rates significantly declined from enrollment to 2 weeks of treatment and further in those who continued to deteriorate by 10 weeks of follow-up. This observation was noticeable regardless of the participants’ randomized arm. Aggregated data from 14 studies reported before optimization of antiretroviral therapy and 12 studies following deferred antiretroviral treatment showed similar survival rates among enrolled and followed-up participants [25]. Reports on persistently high death rates among hospitalized patients and among discharged patients show incident mortality to be associated with the unmasking of immune reconstitution inflammatory syndrome [49]. Relapse of cryptococcal meningitis is common [50,51] and delay in re-hospitalization with fungal relapse or with persistent symptoms [52]. Delayed diagnosis or living with common other opportunistic co-infections with advanced HIV disease [1], severe deficiency of CD4 T cell counts [52,53], and absence of social support while in care [54] were more frequently associated with poor outcomes. Therefore, the diversity of poor survival outcomes treatment-modifying comorbidities associated with these patients confound efficacy outcomes of antifungal regimens.

Clinical trial-related interventions have improved general clinical practice, research, and diagnostic infrastructure in some resource-limited settings where clinical trials were consistently implemented [6,10]. Funding availability is essential in driving alternative diagnostic and treatment interventions in clinical trial settings [9] where patients’ survival improved compared to poorer outcomes in lower tier health settings [6]. With limited antifungal options, the discovery of new antifungal compounds and other drug formulations would benefit survival of patients with cryptococcal meningitis.

## 5. Conclusions

Cryptococcal meningitis, in the context of severe immunodeficiency with advanced HIV disease (CD4 count <100 cells/microliter), delayed diagnosis and treatment, and high prevalence of comorbidities could explain the rates of antifungal regimen efficacy and therefore host survival. The significant deterioration of patients on treatment, with 2-week mortality at 11.5% and 10-week mortality at 26%, implicates unexplained causes or unmet needs of patients beyond antifungal therapy to host-directed therapies or personalized care in improving survival from preventable deaths with cryptococcal meningitis.

## 6. Limitations

The data are limited to the peer-reviewed published literature. The grey literature and unpublished data may have been missed due to un accessibility.

## Figures and Tables

**Table 1.** Studies contributing data and number of participants per trial randomization arm.

Author and Publication Year	Trial Regimen	Trial Arm, N = 1710	10-Week, Survival, *n* (%)	Control Arm, N = 1724; AmB + Flu or (*, **)	10-Week Survival, *n* (%)
Boulware (2023) [34]	IV AmB x2 + oral AmB nanocrystals	40	35 (90%)	41 **	35 (85.4%)
Boulware (2023) [34]	All—oral AmB nanocrystals	40	34 (85%)	41 **	35 (85.4%)
Zhao (2022) [14]	10-week AmB + 5FC	78	70 (89.7%)	50	36 (72%)
Zhao (2022) [14]	10-week VCZ + 5FC	28	24 (85.7%)	50	36 (72%)
Jarvis (2022) [30]	L-AmB 10 mg/kg x1	101	76 (75.2%)	117 **	117 (71.3%)
Ngan NTT, (2021) [23]	AmB + Flu + tamoxifen	24	16 (66.7%)	26	19 (73.1%)
Rhein, (2019) [18]	AmB + fluconazole + sertraline 400 mg	229	113 (50.7%)	231	135 (58.4%)
Katende, (2019) [15]	sertraline + fluconazole	28	6 (21%)	18	11 (61%)
Jarvis, (2019) [29]	Single-dose L-AmB + fluconazole	18	14 (78%)	21	15 (71.4%)
Jarvis, (2019) [29]	2 doses L-AmB + fluconazole	20	17 (85%)	21	15 (71.4%)
Jarvis, (2019) [29]	3 doses L-AmB + fluconazole	20	10 (50%)	21	15 (71.4%)
Molloy (2018) [31]	AmB + 5FC	225	124 (55%)	228	157 (68.9%)
Beardsley (2016) [32]	Dexa + AmB + Flu	224	120 (53.6%)	226	132 (58.4%)
Day J (2013) [24]	AmB	99	55 (56%)	99	66 (67%)
Day J (2013) [24]	AmB + 5FC	100	70 (69%)	99	66 (67%)
Jarvis (2012) [19]	AmB + 5FC + IFN-γ	57	40 (70.2%)	62	42 (67.7%)
Jackson T (2012) [21]	AmB + 5FC	40	25 (62.5%)	39	21 (53.8%)
Loyse (2012) [27]	AmB + 5FC	20	14 (70%)	21	14 (66.7%)
Loyse (2012) [27]	AmB + Flu 1200 mg	22	16 (72.7%)	21	14 (66.7%)
Loyse (2012) [27]	AmB + Vori 300 mg	12	9 (75%)	21	14 (66.7%)
Nussbaum, 2010 [25]	Flu + 5FC	21	12 (57%)	19	8 (42%)
Hamill J.R, (2010) [26]	L-AmB 3 mg/kg	74	64 (86%)	77	68 (88.5%)
Hamill J.R, (2010) [26]	L-AmB 6 mg/kg	85	77 (90.4%)	77	68 (88.5%)
Pappas (2009) [22]	AmB + Flu 400 mg	48	41 (85%)	45	44 (97%)
Pappas (2009) [22]	AmB	47	40 (85%)	45	44 (97%)
Orem J (2005) [20]	Acetazolamide + LPs	10	5 (50%)	8 *	4 (50%)

AmB—amphotericin B deoxycholate, Flu—fluconazole, 5FC—flucytosine, L-Amp—Liposomal amphotericin B deoxycholate, VCZ—voriconazole, Vori—voriconazole, Dexa—dexamethasone, IV—intravenous, 2x—twice, LPs—lumbar punctures, IFN-gamma—interferon gamma, Kg—kilogram, mg—milligram, N—number of enrolled participants per trial randomization arm.

* Acetazolamide.

** Flucytosine.

There was no difference in survival across studies (*p* = 0.715).

## Data Availability

The data generating the manuscript are presented on tables, figures, and in-text citations.
